# Daily Text Messaging for Weight Control Among Racial and Ethnic Minority Women: Randomized Controlled Pilot Study

**DOI:** 10.2196/jmir.2844

**Published:** 2013-11-18

**Authors:** Dori M Steinberg, Erica L Levine, Sandy Askew, Perry Foley, Gary G Bennett

**Affiliations:** ^1^Duke Obesity Prevention ProgramDuke Global Health InstituteDuke UniversityDurham, NCUnited States; ^2^Department of Psychology and NeuroscienceDuke UniversityDurham, NCUnited States

**Keywords:** self-monitoring, mHealth, text messaging, weight loss, black women

## Abstract

**Background:**

Daily self-monitoring of diet and physical activity behaviors is a strong predictor of weight loss success. Text messaging holds promise as a viable self-monitoring modality, particularly among racial/ethnic minority populations.

**Objective:**

This pilot study evaluated the feasibility of a text messaging intervention for weight loss among predominantly black women.

**Methods:**

Fifty obese women were randomized to either a 6-month intervention using a fully automated system that included daily text messages for self-monitoring tailored behavioral goals (eg, 10,000 steps per day, no sugary drinks) along with brief feedback and tips (n=26) or to an education control arm (n=24). Weight was objectively measured at baseline and at 6 months. Adherence was defined as the proportion of text messages received in response to self-monitoring prompts.

**Results:**

The average daily text messaging adherence rate was 49% (SD 27.9) with 85% (22/26) texting self-monitored behavioral goals 2 or more days per week. Approximately 70% (16/23) strongly agreed that daily texting was easy and helpful and 76% (16/21) felt the frequency of texting was appropriate. At 6 months, the intervention arm lost a mean of 1.27 kg (SD 6.51), and the control arm gained a mean of 1.14 kg (SD 2.53; mean difference –2.41 kg, 95% CI –5.22 to 0.39; *P*=.09). There was a trend toward greater text messaging adherence being associated with greater percent weight loss (*r*=–.36; *P*=.08), but this did not reach statistical significance. There was no significant association between goal attainment and text messaging adherence and no significant predictors of adherence.

**Conclusions:**

Given the increasing penetration of mobile devices, text messaging may be a useful self-monitoring tool for weight control, particularly among populations most in need of intervention.

**Trial Registration:**

Clinicaltrials.gov: NCT00939081; http://clinicaltrials.gov/show/NCT00939081 (Archived by WebCite at http://www.webcitation.org/6KiIIcnk1).

## Introduction

Regular self-monitoring of diet and physical activity behaviors is one of the strongest predictors of weight loss success [1,2]. Self-monitoring likely improves weight loss outcomes by activating a host of self-regulatory mechanisms. Regular self-monitoring might enhance self-efficacy, improve accountability, and facilitate awareness of how behaviors affect weight [3]. Weight loss interventions have traditionally used paper-based self-monitoring methods. However, long-term adherence to this approach is poor [1,4], possibly because paper-based methods are time- and labor-intensive, require extensive numeracy and literacy skills, and can be perceived as burdensome [5]. What is concerning is that poor adherence adversely affects weight loss outcomes [1]. In contrast, electronic health (eHealth) self-monitoring approaches include features (eg, prompts, real-time data collection, data-driven feedback, asynchronous communication) that may decrease participant burden and increase adherence [6]. Indeed, evidence does show that electronic self-monitoring via either Web or mobile devices produces greater adherence over traditional paper-and-pencil methods [4,7,8] and improves attitudes toward self-monitoring behaviors [9].

Text messaging shows promise as an alternative eHealth self-monitoring approach [10] and offers several advantages compared to other eHealth modalities (eg, Web, interactive voice response). Data can be entered quickly on nearly all mobile phone platforms, making it portable, proximal to actual behaviors, and more accessible for providing tailored feedback [11]. Additionally, text messaging has been conventionally limited to 160 characters (approximately 15-20 words) per message, limiting the detail and cognitive load that is required. Thus, text messaging may be a viable and sustainable self-monitoring modality.

Text messaging has become ubiquitous [12], particularly among racial/ethnic minority groups [13]. Recent studies show that racial/ethnic minorities are more likely than white individuals to own mobile phones [13]. The high familiarity with and penetration of mobile technologies makes text messaging an ideal intervention platform among these populations. This is notable because we have few interventions that produce clinically meaningful weight loss outcomes among racial/ethnic minority populations [14-16], those with the highest rates of obesity. Black women, in particular, have alarmingly high rates of obesity as compared with other gender and racial/ethnic groups: 59% of black women are obese versus 36% in the general US population [17]. However, little is known about the use of text messaging for self-monitoring weight control behaviors in this population. The purpose of this pilot study was to evaluate the feasibility of daily text messaging for self-monitoring behavioral goals for weight loss among predominantly obese black women. Our secondary aim was to evaluate the effects of the intervention on weight change relative to an education control arm.

## Methods

### Participants

We recruited women aged 25-50 years, with a body mass index (BMI) greater than or equal to 25 kg/m^2^. Other inclusion criteria were willingness to (1) come to all study assessments over 6 months, (2) use a personal cell phone to send and receive up to 5 texts per day for 6 months without compensation for the text messages, and (3) be randomized into either treatment arm. Exclusion criteria included pregnancy or planned pregnancy within the next 6 months and a history of myocardial infarction or stroke within the past 2 years.

### Recruitment and Randomization

We partnered with a nonprofit church-based community wellness organization located in Raleigh, NC to recruit participants. The wellness organization provided the location for enrollment events and aided in recruitment by advertising the study in common spaces and during community meetings and church services. Additional recruitment was conducted in the surrounding community via flyers posted in neighborhood businesses and outreach to adults in the area who had expressed interest in weight loss research trials. Recruitment took place between June and September 2010.

Interested participants visited a study website to complete initial eligibility screening that assessed self-reported age, gender, height, weight, and race (American Indian or Alaska Native, Asian, black or African American, Native Hawaiian or other Pacific Islander, white, or other). Eligible participants were then invited to an in-person enrollment event. Ineligible participants were directed to a website where they could access publicly available weight loss information. At the enrollment events, study staff obtained informed consent and collected baseline anthropometric and survey measures. Participants were then randomized to the intervention arm or the education control arm using a computer-generated algorithm. Because of the pilot nature of this study, participants were re-evaluated at 6 months, with no additional study visits. Participants received a US $35 gift card to a local store as an incentive for participation. The Duke University Institutional Review Board approved this study.

### Intervention Arm

#### Overview

The intervention (Shape Plan) included daily tracking of tailored behavior change goals through text messaging and personalized daily and weekly feedback via text messaging and email, respectively. Participants also received information sheets about behavioral goals, a pedometer, 2 face-to-face group sessions, and a skills training DVD.

#### Behavior Change Goals

Behavior change goals were determined using the interactive obesity treatment approach (iOTA) [18-20], a theory-based approach whereby participants are assigned an individualized set of routine lifestyle behavior change goals and directed to change them to create an energy deficit sufficient to produce weight change. Lifestyle behavior change goals are assigned based on an algorithm that considers participants’ need and self-efficacy around changing behaviors, as well as the expected caloric deficit. The iOTA library (Textbox 1) contains 12 obesogenic behaviors framed as goals to create a caloric deficit for weight loss (eg, no sugary drinks, walking 7000 steps per day, no fast food) that were selected based on their (1) empirical support, (2) population relevance, (3) ease of self-monitoring, and (4) concreteness. Participants were assigned new goals at 3 months to introduce novelty, maintain motivation, and facilitate goal mastery.

At baseline, the iOTA algorithm ranked behavior change goals for intervention participants. Participants were instructed to self-monitor the 2 top goals daily for 12 weeks. All intervention participants also received a walking goal of at least 7000 steps every day. The physical activity goal increased based on participants’ performance, up to 10,000 steps per day. The survey was re-administered at 3 months and updated goals were assigned using the same algorithm.

**List of iOTA behavioral goals.**

- Walk 7000/8000/10,000 steps every day
- No sugary drinks
- Eat 5 or more fruits and vegetables every day
- No chips, cookies, or candy
- Switch to low-fat dairy
- No fast food
- Eat breakfast every day
- Watch no more than 2 hours of TV every day
- No fried food
- No snacks or dessert after dinner
- No more than 1 alcoholic drink per day
- Eat red meat no more than once a week

#### Information Sessions

At the baseline enrollment event, Shape Plan participants received a group-based orientation to the intervention led by community health educators experienced in delivering information on weight control. The orientation included a review of the iOTA goals, calorie balance, a demonstration of the text messaging self-monitoring and feedback, and an action planning session. Goal setting and text messaging monitoring began the following day. At 3 months, in an effort to reduce the number of face-to-face meetings, participants received a set of videos with skills training information on topics such as healthy eating patterns, eating cues, recognizing hunger, problem solving to meet goals, goal setting, exercise tips, and safety and action planning for the upcoming Shape Plan goals. At 6 months, participants received another hour-long group face-to-face session that focused on problem solving, assessment of overall progress, and tips for maintaining behavior changes.

#### Self-Monitoring and Feedback Through Text Messaging

**Figure 1:**
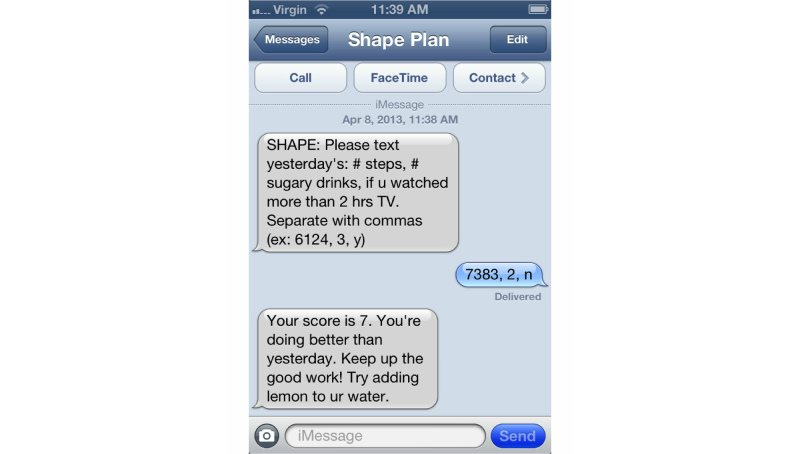
Screenshot of self-monitoring via text message with feedback.

The text messaging protocol included 1 daily morning text message at 8:00 am, which asked participants to report performance on their goals from the previous day (eg, “How many steps did you walk yesterday?”;Figure 1). Although immediate self-monitoring was encouraged, participants could respond any time until 7:59 am the next day. Any responses received within the 24-hour window after the outbound text message was sent were counted as a successful self-monitoring response. A score was assigned to each goal based on self-monitoring data received from participants. For example, if a participant was working on the no sugary drinks goal, a score of 10 was given if the participant entered 0 drinks that day, a score of 5 if the participant reported 2 drinks, and a score of 1 if ≥5 drinks was entered. Conversely, if the goal was to eat 5 or more fruits and vegetables, a score of 10 was given if the participant entered ≥5 fruits and vegetables for that day, and a score of 1 if a participant reported eating 0 fruits and vegetables that day. A summary score was then calculated for all 3 goals together, with higher scores indicative of high overall goal attainment (range 1-10). A feedback message was sent via an automated system based on the summary score, along with relative feedback based on the previous day of self-monitoring (eg, “You’re doing better than yesterday—great job!” or “You did worse than last time. Let’s turn this slip around”) and specific tips on how to change low-scoring goals (eg, “Try flavored seltzer water instead of regular soda” or “Try sliced bell peppers as a snack”). Messaging content was based on previous studies conducted using the iOTA approach in this population [19-22]. Rigorous testing of the logic was completed before the start of the intervention, and continuous quality checks were performed to ensure fidelity to protocol.

Additionally, Shape Plan participants received a weekly automated email on Sundays with a summary of their progress. Participants with at least 3 days of self-monitoring data received a weekly email with personalized feedback that included a summary of goal attainment and a graph of progress over the previous week. For participants with low adherence (3 or fewer texts in 1 week), the email did not include a summary, but rather acted as a prompt to improve adherence (eg, “We only received 2 text messages from you this week. In order for you to be most successful losing weight in Shape Plan, you should track your numbers and send us a text every day”).

### Education Control Arm

To control for contact and isolate the behavior change goals, self-monitoring via text messaging and feedback, participants randomized to the education control arm received (1) 2 in-person group education sessions, one at baseline and another at 6 months; (2) a set of videos at 3 months that covered topics such as healthy eating patterns, eating cues, recognizing hunger, exercise recommendations, and how to read a nutrition facts food label; (3) pedometers; and (4) a “prescription” to walk 10,000 steps per day. Control arm participants received no text messaging during the study period, but had the option to receive a 3-month version of the text messaging intervention after the study was complete.

### Measures

#### Demographic Characteristics

At baseline, a variety of sociodemographic variables were collected through an online survey to characterize the sample, including age, race/ethnicity, household income, education, marital status, and employment.

#### Self-Monitoring Adherence

A study database collected and stored text messaging self-monitoring data. Adherence was defined as the proportion of self-monitoring texts received of the number expected over the 6-month period (N=167). We examined total adherence and adherence by study week.

#### Anthropometrics

At baseline and 6 months, trained staff measured participant heights to the nearest 0.1 cm using a calibrated stadiometer (Seca 214). Weights were measured to the nearest 0.1 kg using an electronic scale (Seca Model 876) [23].

#### Program Satisfaction

At 6 months, intervention participants completed a 23-item online questionnaire to assess intervention satisfaction. Using a 4-point Likert scale with response options ranging from strongly agree (=4) to strongly disagree (=1), participants rated whether they found daily self-monitoring through text messaging to be easy, helpful overall, helpful for increasing daily steps, and important. Similarly, participants reported whether daily text messages were the appropriate frequency and whether they were satisfied with the feedback received via text messaging.

#### Statistical Analyses

We used chi-square tests and *t* tests to examine differences in baseline characteristics between study arms. Similar tests were used to describe the average rate of text messaging adherence and the proportion of participants who achieved various thresholds of self-monitoring adherence. We used intent-to-treat analyses, with baseline weight carried forward for missing data. The *t* tests were used to examine absolute weight change, percent weight loss, and BMI change between study arms, and ANOVA was conducted to examine goal attainment and weight change across tertiles of self-monitoring adherence. Pearson correlation tests were conducted to examine the overall association between text messaging adherence and goal attainment and weight loss. Analyses were conducted using SPSS ver 19 for Mac (IBM Corp, Armonk, NY, USA). All tests were 2-tailed and an alpha level <.05 was used to assess statistical significance.

## Results

### Enrollment and Retention

**Figure 2:**
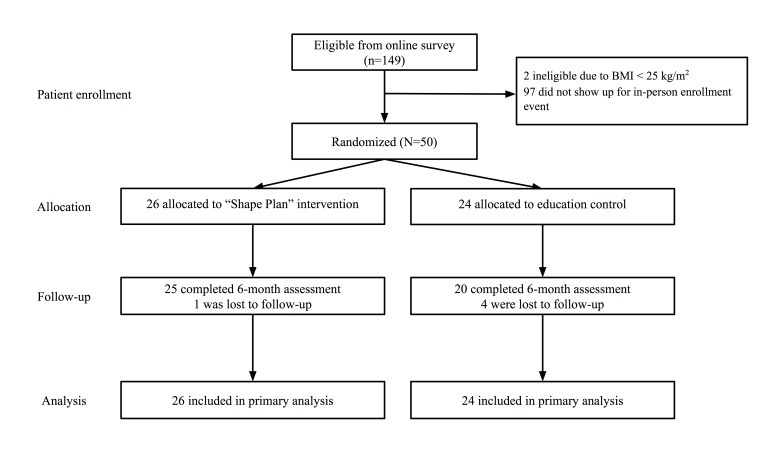
Flowchart of participant enrollment and retention in the study.

Figure 2 outlines the study enrollment and retention flow. A total of 149 individuals were deemed eligible from the online screener. The total number screened via the study website is not known. Of those initially eligible, 2 were ineligible because of BMI and 97 did not attend the enrollment event. In all, 50 participants were randomized to either the Shape Plan intervention (n=26) or the control (n=24) arm. At 6 months, 90% (45/50) of participants attended the 6-month follow-up assessment visit. There were no significant differences in attrition between study arms.

### Baseline Characteristics

Mean age of participants was 38.3 years (SD 8.2). Participants were obese (BMI mean 35.8 kg/m^2^, SD 6.1), and had an average weight of 99.1 kg (SD 20.0) (Table 1). Participants were predominantly black (82%, 41/50), employed (82%, 41/50), and college educated (64%, 32/50). Approximately one-third of the sample (32%, 16/50) had an annual income less than US $40,000 and 50% (25/50) were married or living with a partner. There were no significant differences between arms with regard to baseline sociodemographic characteristics.

### Text Messaging Adherence

**Figure 3:**
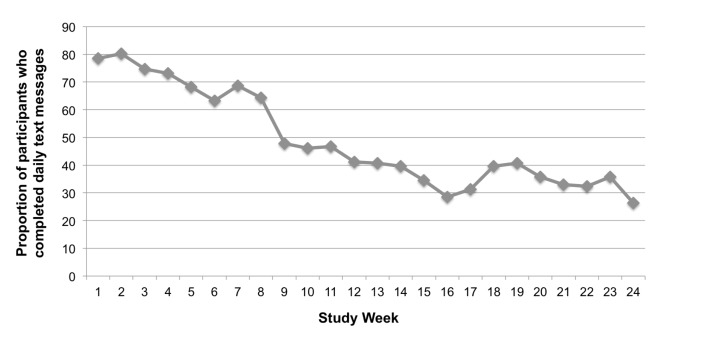
Adherence to daily self-monitoring via text message by study week (n=26).

Figure 3 shows the text messaging self-monitoring completion rates over time by study week. Among all randomized intervention participants (n=26), the daily text messaging adherence rate was 49% (SD 28, IQR 27%-78%). Most participants (58%, 15/26) texted at least 3 days each week and 85% (22/26) texted at least 2 days per week, on average. There were no significant predictors of adherence. During the study, 8 participants demonstrated nonusage attrition [24], requesting to cease intervention participation before the end of the trial for a host of reasons (eg, cost of text messaging, change in phone service, not interested in continued participation). Excluding these 8 participants, the adherence rate was 54% (SD 25), and we similarly found no significant predictors of adherence.

The mean daily goal attainment score over the 6-month intervention period was 6.3 (SD 2.8, IQR 4.0-8.2), indicating moderate-high adherence to behavioral goals. Similarly, the mean number of steps reported was 4994 (SD 2741, IQR 3016-6489). There was no significant correlation between average goal attainment score and text messaging adherence (*r*=.24; *P*=.25) or mean number of steps reported and text messaging adherence (*r*=.33; *P*=.11). However, when examining tertiles of adherence, there was a trend toward greater adherence being associated with greater step counts, although this did not reach statistical significance (tertile 1: mean 3958, SD 2698; tertile 2: mean 4174, SD 1902; tertile 3: mean 6481, SD 2895; *P*=.09).

**Table 1:** Baseline characteristics of participants by study arm.

Characteristic		Total (N=50)	Control (n=24)	Shape Plan (n=26)
**Marital status, n (%)**				
	Married or living with a partner	25 (50)	9 (38)	16 (62)
	Divorced/separated or never married	25 (50)	15 (62)	10 (38)
**Employment, n (%)**				
	Employed	41 (82)	19 (79)	22 (85)
	Unemployed	9 (18)	5 (21)	4 (15)
**Education, n (%)**				
	4 year college degree or higher	32 (64)	15 (62)	17 (65)
	Less than a 4 year college degree	18 (36)	9 (38)	9 (35)
**Race/ethnicity, n (%)**				
	Non-Hispanic black	41 (82)	18 (75)	23 (88)
	Non-Hispanic other	9 (18)	6 (25)	3 (12)
**Household income (US $), n (%)**				
	<40,000	16 (32)	9 (38)	7 (27)
	40,000-69,999	18 (36)	7 (29)	11 (42)
	≥70,000	16 (32)	8 (33)	8 (31)
Age (years), mean (SD)		38.3 (8.2)	39.0 (9.0)	37.6 (7.4)
Weight (kg), mean (SD)		99.1 (20.0)	96.0 (23.1)	102.0 (16.6)
BMI (kg/m^2^), mean (SD)		35.8 (6.1)	34.6 (5.8)	36.9 (6.2)

### Intervention Satisfaction

At 6 months, most participants strongly agreed that texting was easy (70%, 16/23) and helpful (68%, 15/22), and 76% (16/21) either somewhat or strongly agreed that the text messages helped them increase the number of daily steps walked. Almost three-quarters of participants (71%, 15/21) reported that it took less than 3 minutes to reply to texts and most (79%, 15/19) responded to prompts for self-monitoring via text message in the morning. More than half (57%, 12/21) felt that receiving daily texts was very important and approximately three-quarters of participants (76%, 16/21) felt the frequency of texting was appropriate. Most (82%, 18/22) were satisfied with the feedback content they received via text messaging and 62% (13/21) felt the feedback was very important.

### Weight Change

Table 2 highlights changes in weight and BMI between study arms. At 6 months, using intent-to-treat analyses (N=50), participants randomized to the control arm gained a mean of 1.14 kg (SD 2.53), whereas intervention participants lost a mean of 1.27 kg (SD 6.51). The mean difference was –2.41 kg (95% CI –5.22 to 0.39; *P*=.09). This equates to a percent weight loss of 0.97% (SD 5.35) in the intervention arm, which was marginally higher than a gain of 1.32% (SD 2.77) in the control arm (mean difference –2.29, 95% CI –4.74 to 0.16; *P*=.06). There were no significant differences between arms with regard to changes in BMI. Similar results were found when we restricted the sample to study completers only (n=45). There were no significant correlations between adherence to text messaging and change in weight or BMI. However, there was a trend toward greater text messaging adherence being associated with greater percent weight loss (*r* = –.36; *P*=.08), but this did not reach statistical significance. Similarly, there were no differences in weight outcomes by tertiles of text messaging adherence (data not shown).

**Table 2:** Change in weight and body mass index (BMI) between baseline and 6 months by study group (N=50).

Weight change variables	Control, mean (SD) (n=24)	Shape Plan, mean (SD) (n=26)	Mean difference (95% CI)	*P* value
Change in weight (kg)	1.14 (2.53)	–1.27 (6.51)	–2.41 (–5.22, 0.39)	.09
Percent weight loss (%)	1.32 (2.77)	–0.97 (5.35)	–2.29 (–4.70, 0.12)	.06
Change in BMI (kg/m^2^)	0.42 (0.90)	–0.47 (2.42)	–0.89 (–1.93, 0.15 )	.09

## Discussion

### Principal Findings

In this pilot study, we found that daily text messaging for behavioral self-monitoring is both feasible and positively perceived. Approximately half of participants were fully adherent to daily self-monitoring through text messages during the 6-month study and 84% stayed active in the intervention throughout the study period. In contrast to previous studies that included paper-based self-monitoring modes [5], most participants felt that text messaging was helpful, easy, and important for achieving their behavior change goals. Text messaging has major advantages relative to other approaches. It has high familiarity and a greater potential for broad reach, particularly among racial/ethnic minority populations. Despite the intervention having only a marginal effect on weight change, these results indicate that text messaging may be a viable way to collect self-monitoring data and deliver intervention feedback and skills training.

The eHealth and mobile health (mHealth) approaches to self-monitoring offer numerous advantages over traditional approaches. Self-monitoring via text messaging is cheaper, easier to program, and more proximal to behavior changes as compared to paper-based and Web-based self-monitoring. Both eHealth and mHealth self-monitoring strategies seem not to exhibit the same steep decline typically seen in self-monitoring adherence [1,6,11] particularly when feedback is provided in response to self-monitoring [4,25]. The current pilot study provided immediate feedback in response to text messaging self-monitoring and found that most participants reported high satisfaction with this feedback. Providing feedback via text messaging may offer particular benefits over other modalities. However, feedback can only be sent when participants provide self-monitoring data; thus, finding ways to enhance adherence remains important.

What participants are self-monitoring may be as important to adherence as how they are self-monitoring. Typical self-monitoring behaviors include detailed reports of food intake, including portion size, and calorie and fat content. Although effective, self-monitoring of this type exhibits poor adherence [4,6]. Our pilot study, in contrast, included simple and discrete self-monitoring of behavior change goals associated with weight loss (eg, no sugary drinks, no fried food). Self-monitoring of specific behaviors rather than detailed dietary records may be less burdensome and more fitting for delivery via text messaging. Indeed, we found high satisfaction with our approach, adherence rates comparable to Web-based self-monitoring approaches [7,26], and a trend toward a positive association between adherence and weight loss. Whether text messaging for self-monitoring is more effective for weight change compared to other eHealth or paper-based approaches is not clear. Trials examining the comparative efficacy of these different modes are necessary.

To date, our weight loss outcomes are similar to those of other text messaging trials [27-29]. Most of these trials used text messaging to deliver skills training information and feedback via outbound messages. We are aware of only 1 trial that used text messaging to monitor behaviors and provide feedback. In their Text4Diet trial, Shapiro et al [29] tested an intervention that asked participants to self-monitor daily pedometer steps and weekly body weight. The intervention sent up to 4 text messages per day for 12 months that included personalized tips for eating behaviors, reminders, educational facts, motivational messages, knowledge-based questions, and feedback in response to self-monitoring data. After 12 months, the intervention arm lost 1.65 kg, but there were no significant differences between treatment arms.

Our pilot study used text messaging to collect self-monitoring data on diet and physical activity behaviors, but we did not gather data on body weight or include any type of coaching support. This may have affected the weight losses achieved because body weight self-monitoring has been shown to be effective in the absence of self-monitoring of other behaviors [30]. Haapala and colleagues [28] emphasized calorie counting and used body weight to adjust calorie goals, which may have resulted in larger weight loss outcomes. By contrast, the Shape Plan study focused on engagement in behaviors known to produce a caloric deficit (eg, switching from high-fat dairy to low-fat dairy) rather than calorie counting to achieve weight loss. Long-term, we hypothesize that our approach may have greater potential for sustainability because calorie counting is cognitively complex and may not be suitable for populations with lower levels of education. However, this may come with some cost to the magnitude of weight loss.

Black women have the highest prevalence of obesity compared to any other group [17] and achieving clinically meaningful weight loss among this group has been particularly challenging [16]. Across numerous studies, black women achieved smaller weight losses compared to other groups and most did not lose more than 4 kg [15]. These results indicate a need for new obesity treatment approaches for black women that are relevant, effective, and sustainable. In the current study, we found that there was a trend toward a small weight loss among intervention participants and a small weight gain among control participants. Although our study focused on weight loss, this low-intensity approach may be helpful for staving off premenopausal weight gain that is common among black women and higher than other racial/ethnic groups [31,32]. Given the high satisfaction with text messaging for self-monitoring and comparable adherence ranges, future research might include this modality along with brief coaching calls in an effort to enhance weight loss outcomes.

Additionally, mobile phone use is high among blacks, and black individuals are more likely than white individuals are to use mobile phones to look for health or medical information [33]. Previous studies testing text messaging as a self-monitoring tool for weight loss included predominantly white samples with greater sociodemographic advantage. One exception is a recent trial that was short in duration, but found a positive association between text messaging for self-monitoring and walking among an older, black, and predominantly female sample [34]. These findings are consistent with our study and further confirm that this modality is feasible for behavior change, with the potential for broad reach.

### Strengths and Limitations

This study has several strengths. Most of our sample (82%) were black women, which is a group typically underrepresented in weight control research. Although a strength of our study, the findings may not generalize to other populations and settings. The goal of this study was to test the feasibility of text messaging for self-monitoring through a low-intensity weight loss intervention among an understudied population. We isolated the impact of text messaging self-monitoring along with feedback with a control arm that received comparable group information sessions and the use of a pedometer. This study also has some limitations worth mentioning. A few participants (n=8) experienced barriers to participation, such as cost of text messaging and disconnected cell phone service, or they were no longer interested in participating. Although providing phones and/or text messaging plans may have enhanced our adherence rates, the use of personal cell phones provides insight into the “real world” feasibility of text messaging for weight control and improves the generalizability of the intervention.

Although comparable to other eHealth weight control interventions, higher adherence rates are needed to produce greater weight losses. This pilot study was low intensity and did not include any contact with study staff outside the assessment visits. Our main goal was to assess the feasibility of using text messaging for self-monitoring behavioral goals among a predominantly racial/ethnic minority population. To enhance adherence, future studies using this approach might also include daily tracking of weight and provide feedback on weight loss progress via text message. More frequent skills training through monthly videos may also boost adherence rates. Including elements of accountability and support (eg, monthly coaching calls with a lifestyle counselor) can also be an effective way to enhance adherence, but including these components will increase intensity and make it more difficult to ascertain the independent effects of the text messaging. Given that this was a pilot study, the small sample size limited our power to assess whether this intervention led to significantly greater weight loss as compared to an education control arm. Future studies should examine the efficacy of this approach with a larger sample size, longer duration, and multiple measures throughout the study period.

### Conclusions

Text messaging holds promise as a self-monitoring modality for weight control, particularly among groups most at risk for obesity-related morbidities. Given that the majority of evidence indicates that greater adherence leads to better outcomes, our study suggests that mHealth-based approaches to self-monitoring may enhance engagement and reduce the burdens commonly associated with other modes. Our intervention was relatively low intensity and has greater potential for dissemination compared to higher intensity interventions. As technology penetration increases in the target population, the use of this modality will become increasingly relevant and helpful as an intervention tool for weight control.

## Multimedia Appendix 1

CONSORT-EHEALTH checklist V1.6.2 [35].

## Abbreviations

BMI: body mass index
eHealth: electronic health
iOTA: interactive obesity treatment approach
mHealth: mobile health
