# Supplementary material for: Daily Text Messaging for Weight Control Among Racial and Ethnic Minority Women: Randomized Controlled Pilot Study
Source: J Med Internet Res. 2013 Nov 18;15(11):e244. doi: 10.2196/jmir.2844 (PMC3841371; doi:10.2196/jmir.2844)
Supplement: Supplementary file 1 [file jmir_v15i11e244_app1.pdf]

CONSORT-EHEALTH Checklist V1.6.2 Report

(based on CONSORT-EHEALTH V1.6), available at [http://tinyurl.com/consort-ehealth-v1-6].

2844

Date completed

7/22/2013 16:45:31

by

Steinberg

Daily text messaging for weight control among racial/ethnic minority women: A randomized-controlled pilot study

TITLE

1a-i) Identify the mode of delivery in the title

"Daily text messaging for weight control among racial/ethnic minority women"

1a-ii) Non-web-based components or important co-interventions in title

no

1a-iii) Primary condition or target group in the title

"...among racial/ethnic minority women"

ABSTRACT

1b-i) Key features/functionalities/components of the intervention and comparator in the METHODS section of the ABSTRACT

"Methods: Fifty obese women were randomized to a 6-month intervention that included daily text messages for self-monitoring tailored behavioral goals (e.g., no sugary drinks, 10,000 steps per day) along with brief feedback and tips using a full automated-system or an education control arm. Weight was measured at baseline and 6 months. Adherence was defined as the proportion of text messages received in response to self-monitoring prompts."

1b-ii) Level of human involvement in the METHODS section of the ABSTRACT

"Methods: Fifty obese women were randomized to a 6-month intervention that included daily text messages for self-monitoring tailored behavioral goals (e.g., no sugary drinks, 10,000 steps per day) along with brief feedback and tips using a full automated-system or an education control arm. Weight was measured at baseline and 6 months. Adherence was defined as the proportion of text messages received in response to self-monitoring prompts."

1b-iii) Open vs. closed, web-based (self-assessment) vs. face-to-face assessments in the METHODS section of the ABSTRACT

"Weight was objectively measured at baseline and 6 months. "

1b-iv) RESULTS section in abstract must contain use data

"Results: The average daily text messaging adherence rate was 49.0% ± 27.9%; 84.6% self-monitored behavioral goals ≥2 days per week. Almost 70% strongly agreed that daily texting was easy and helpful and 76.2% felt the frequency of texting was appropriate. At 6 months, the intervention arm lost 1.27±6.51kg, and the control arm gained 1.14±2.53kg [mean difference: -2.41kg (-5.22, 0.39); p= 0.09]. There was a trend towards greater text messaging adherence being associated with greater percent weight loss (r = -.363; p=.08)."

1b-v) CONCLUSIONS/DISCUSSION in abstract for negative trials

"Conclusion: Given the increasing penetration of mobile devices, text messaging may be a useful self-monitoring tool for weight control, particularly among populations most in need of intervention."

INTRODUCTION

2a-i) Problem and the type of system/solution

"Text messaging has become ubiquitous,[12] particularly among racial/ethnic minority groups.[13] Recent studies show that racial/ethnic minorities are more likely than Whites to own mobile phones.[13] The high familiarity with, and penetration of mobile technologies makes text messaging an ideal intervention platform among these populations. This is notable because we have few interventions that produce clinically meaningful weight loss outcomes among racial/ethnic minority populations,[14-16] those with the highest rates of obesity. Black women, in particular, have alarmingly high rates of obesity as compared to other gender and racial/ethnic groups; 59% of Black women are obese as compared to 36% in the general U.S. population.[17] However, little is known about the use of text messaging for self-monitoring weight control behaviors in this population. "

2a-ii) Scientific background, rationale: What is known about the (type of) system

"In contrast, electronic health (eHealth) self-monitoring approaches include features (e.g. prompts, real-time data collection, data driven feedback, asynchronous communication) that may decrease participant burden and increase adherence.[6] Indeed, evidence does show that electronic self-monitoring either via web or mobile devices produces greater adherence over traditional paper and pencil methods,[4, 7, 8] and improves attitudes towards self-monitoring behaviors.[9]

Text messaging shows promise as an alternative eHealth self-monitoring approach[10] and offers several advantages compared to other eHealth modalities (e.g., web, interactive voice response). Data can be entered quickly on nearly all mobile phone platforms, making it portable, proximal to actual behaviors, and more accessible for providing tailored feedback.[11] Additionally, text messaging has been conventionally limited to 160 characters (approximately 15-20 words) per message, limiting the detail and cognitive load that is required. Thus, text messaging may be a viable and sustainable self-monitoring modality."

METHODS

3a) CONSORT: Description of trial design (such as parallel, factorial) including allocation ratio

"The purpose of this pilot study was to evaluate the feasibility of daily text messaging for self-monitoring behavioral goals for weight loss among predominantly obese Black women. Our secondary aim was to evaluate the effects of the intervention on weight change, relative to an education control arm."

3b) CONSORT: Important changes to methods after trial commencement (such as eligibility criteria), with reasons

n/a - We did not have any changes.

3b-i) Bug fixes, Downtimes, Content Changes

no - we do not have documented reports on this.

4a) CONSORT: Eligibility criteria for participants

""We recruited women, aged 25-50, with body mass index (BMI) greater than or equal to 25 kg/m2. Other inclusion criteria were: willingness to come to all study assessments over 6 months, use of a personal cell phone to send and receive up to five texts per day for 6 months without compensation for text messages, and to be randomized into either treatment arm. Exclusion criteria included pregnancy or planned pregnancy within the next 6 months and a history of myocardial infarction or stroke within the past 2 years.""

4a-i) Computer / Internet literacy

no - we do not have reports on this.

4a-ii) Open vs. closed, web-based vs. face-to-face assessments:

"Interested participants visited a study website to complete initial eligibility screening that assessed self-reported age, gender, height, weight and race. Eligible participants were then invited to an in-person enrollment event. Ineligible participants were directed to a website where they could access publicly available weight-loss information. At the enrollment events, study staff obtained informed consent, and collected baseline anthropometric and survey measures."

4a-iii) Information giving during recruitment
"We partnered with a non-profit church-based community wellness organization located in Raleigh, NC to recruit participants. The wellness organization provided the location for enrollment events and aided in recruitment by advertising the study in common spaces and during community meetings and church services. Additional recruitment was conducted in the surrounding community via flyers posted in neighborhood businesses and outreach to adults in the area who had expressed interest in weight loss research trials. Recruitment took place between June and September 2010."
4b) CONSORT: Settings and locations where the data were collected
"We partnered with a non-profit church-based community wellness organization located in Raleigh, NC to recruit participants. The wellness organization provided the location for enrollment events and aided in recruitment by advertising the study in common spaces and during community meetings and church services. "
4b-i) Report if outcomes were (self-)assessed through online questionnaires
"At baseline, a variety of sociodemographic variables were collected via an online survey to characterize the sample, including age, race/ethnicity, household income, education, martial status, and employment."

"A study database collected and stored text messaging self-monitoring data."

"At 6 months, intervention participants completed a 23-item onine questionnaire to assess intervention satisfaction"
4b-ii) Report how institutional affiliations are displayed
n/a
5) CONSORT: Describe the interventions for each group with sufficient details to allow replication, including how and when they were actually administered
5-i) Mention names, credential, affiliations of the developers, sponsors, and owners
"Behavior change goals
Behavior change goals were determined using iOTA (Interactive Obesity Treatment Approach);[18-20] a theory-based approach whereby participants are assigned an individualized set of routine lifestyle behavior change goals and directed to change them in order to create an energy deficit sufficient to produce weight change. Lifestyle behavior change goals are assigned based on an algorithm that considers participants' need and self-efficacy around changing behaviors, as well as the expected caloric deficit. The iOTA library (Table 1) contains 12 obesogenic behaviors framed as goals to create a caloric deficit for weight loss (e.g., no sugar sweetened beverages, walking 7,000 steps per day, no fast food) that were selected based on their: 1) empirical support; 2) population relevance; 3) ease of self-monitoring; and 4) concreteness. Participants were assigned new goals at three months to introduce novelty, maintain motivation, and facilitate goal mastery.
At baseline, the iOTA algorithm ranked behavior change goals for intervention participants. Participants were instructed to self-monitor the two top goals daily for 12 weeks. All intervention participants also received a walking goal of at least 7,000 steps every day. The physical activity goal increased based on participant's performance, up to 10,000 steps per day. The survey was re-administered at 3 months and updated goals were assigned using the same algorithm.
Information sessions
At the baseline enrollment event, intervention participants received a group-based orientation to the intervention led by community health educators experienced in delivering information on weight control. The orientation included a review of the iOTA goals, calorie balance, a demonstration of the text messaging self-monitoring and feedback, and an action planning session. Goal setting and text messaging monitoring began the following day. At 3 months, participants received a DVD with skills training information, and at 6 months, participants received another hour-long group face-to-face session that focused on problem solving, assessment of overall progress, and tips for maintaining behavior changes.
Self-monitoring and feedback via text messaging
The text messaging protocol (Figure 1) included one daily morning text message at 8:00 am, which asked participants to report performance on their goals from the previous day (e.g., "how many steps did you walk yesterday?"). Although immediate self-monitoring was encouraged, participants could respond any time until 7:59am the next day. Any responses received within the 24-hour window after the outbound text message was sent were counted as a successful self-monitoring response. Two scores were then calculated: one for each of the three discrete behavior change goals, and a summary score that considered all three goals simultaneously. A feedback message was then sent via an automated system based on the summary score, along with relative feedback based on the previous week of self-monitoring (e.g., "you did better than last time – great job!" or "you did worse than last time. Let's turn this slip around") and specific tips on how to change low-scoring goals (e.g., "try flavored seltzer water instead of regular soda" or "try sliced bell peppers as a snack"). Messaging content was based on previous studies conducted using the iOTA approach in this population.[19-22] Rigorous testing of the logic was completed prior to the start of the intervention, and continuous quality checks were performed to ensure fidelity to protocol.
All intervention participants received a weekly automated email with a summary of their progress on Sundays. Participants with at least three days of self-monitoring data received a weekly email with personalized feedback that included a summary of goal attainment and a graph of progress over the previous week. For participants with low adherence (three or fewer texts in one week), the email did not include a summary, but rather acted as a prompt to improve adherence (e.g., "We only received 2 text messages from you this week. In order for you to be most successful losing weight in Shape, you should track your numbers and send us a text every day")."
5-ii) Describe the history/development process
"Behavior change goals were determined using iOTA (Interactive Obesity Treatment Approach);[18-20] a theory-based approach whereby participants are assigned an individualized set of routine lifestyle behavior change goals and directed to change them in order to create an energy deficit sufficient to produce weight change. "

"Messaging content was based on previous studies conducted using the iOTA approach in this population.[19-22] "
5-iii) Revisions and updating
n/a
5-iv) Quality assurance methods
"Rigorous testing of the logic was completed prior to the start of the intervention, and continuous quality checks were performed to ensure fidelity to protocol"
5-v) Ensure replicability by publishing the source code, and/or providing screenshots/screen-capture video, and/or providing flowcharts of the algorithms used
Figure 1
5-vi) Digital preservation
n/a
5-vii) Access

"Other inclusion criteria were: willingness to come to all study assessments over 6 months, use of a personal cell phone to send and receive up to five texts per day for 6 months without compensation for text messages, and to be randomized into either treatment arm. "

"Participants received a $35 gift card to a local store as an incentive for participation."

"Interested participants visited a study website to complete initial eligibility screening that assessed self-reported age, gender, height, weight and race. Eligible participants were then invited to an in-person enrollment event. "

5-viii) Mode of delivery, features/functionalities/components of the intervention and comparator, and the theoretical framework

"The text messaging protocol (Figure 1) included one daily morning text message at 8:00 am, which asked participants to report performance on their goals from the previous day (e.g., "how many steps did you walk yesterday?"). Although immediate self-monitoring was encouraged, participants could respond any time until 7:59am the next day. Any responses received within the 24-hour window after the outbound text message was sent were counted as a successful self-monitoring response. Two scores were then calculated: one for each of the three discrete behavior change goals, and a summary score that considered all three goals simultaneously. A feedback message was then sent via an automated system based on the summary score, along with relative feedback based on the previous week of self-monitoring (e.g., "you did better than last time – great job!" or "you did worse than last time. Let's turn this slip around") and specific tips on how to change low-scoring goals (e.g., "try flavored seltzer water instead of regular soda" or "try sliced bell peppers as a snack"). Messaging content was based on previous studies conducted using the iOTA approach in this population.[19-22] Rigorous testing of the logic was completed prior to the start of the intervention, and continuous quality checks were performed to ensure fidelity to protocol.

All intervention participants received a weekly automated email with a summary of their progress on Sundays. Participants with at least three days of self-monitoring data received a weekly email with personalized feedback that included a summary of goal attainment and a graph of progress over the previous week. For participants with low adherence (three or fewer texts in one week), the email did not include a summary, but rather acted as a prompt to improve adherence (e.g., "We only received 2 text messages from you this week. In order for you to be most successful losing weight in Shape, you should track your numbers and send us a text every day")."

5-ix) Describe use parameters

n/a

5-x) Clarify the level of human involvement

"At the baseline enrollment event, intervention participants received a group-based orientation to the intervention led by community health educators experienced in delivering information on weight control. The orientation included a review of the iOTA goals, calorie balance, a demonstration of the text messaging self-monitoring and feedback, and an action planning session. Goal setting and text messaging monitoring began the following day. At 3 months, participants received a DVD with skills training information, and at 6 months, participants received another hour-long group face-to-face session that focused on problem solving, assessment of overall progress, and tips for maintaining behavior changes."

5-xi) Report any prompts/reminders used

"The text messaging protocol (Figure 1) included one daily morning text message at 8:00 am, which asked participants to report performance on their goals from the previous day (e.g., "how many steps did you walk yesterday?"). Although immediate self-monitoring was encouraged, participants could respond any time until 7:59am the next day. Any responses received within the 24-hour window after the outbound text message was sent were counted as a successful self-monitoring response."

"For participants with low adherence (three or fewer texts in one week), the email did not include a summary, but rather acted as a prompt to improve adherence (e.g., "We only received 2 text messages from you this week. In order for you to be most successful losing weight in Shape, you should track your numbers and send us a text every day")."

5-xii) Describe any co-interventions (incl. training/support)

n/a

6a) CONSORT: Completely defined pre-specified primary and secondary outcome measures, including how and when they were assessed

"A study database collected and stored text messaging self-monitoring data. Adherence was defined as the proportion of self-monitoring texts received of the number expected (n=167). We examined total adherence as well as adherence by study week.

Anthropometrics

At baseline and 6 months, trained staff measured participant heights to the nearest 0.1 cm using a calibrated stadiometer; (Seca 214) and weights were measured to the nearest 0.1 kg using an electronic scale (Seca Model 876).[23]

Program satisfaction

At 6 months, intervention participants completed a 23-item onine questionnaire to assess intervention satisfaction. Using a 4-point Likert scale with response options ranging from strongly agree to strongly disagree, participants rated whether they found daily self-monitoring via text messages to be easy, helpful overall, helpful for increasing daily steps, and important. Similarly, participants reported whether daily text messages were the appropriate frequency and whether they were satisfied with the feedback received via text messaging."

6a-i) Online questionnaires: describe if they were validated for online use and apply CHERRIES items to describe how the questionnaires were designed/deployed

n/a

6a-ii) Describe whether and how "use" (including intensity of use/dosage) was defined/measured/monitored

"A study database collected and stored text messaging self-monitoring data. Adherence was defined as the proportion of self-monitoring texts received of the number expected (n=167). We examined total adherence as well as adherence by study week"

6a-iii) Describe whether, how, and when qualitative feedback from participants was obtained

"At 6 months, intervention participants completed a 23-item onine questionnaire to assess intervention satisfaction. Using a 4-point Likert scale with response options ranging from strongly agree to strongly disagree, participants rated whether they found daily self-monitoring via text messages to be easy, helpful overall, helpful for increasing daily steps, and important. Similarly, participants reported whether daily text messages were the appropriate frequency and whether they were satisfied with the feedback received via text messaging."

6b) CONSORT: Any changes to trial outcomes after the trial commenced, with reasons

n/a

7a) CONSORT: How sample size was determined

7a-i) Describe whether and how expected attrition was taken into account when calculating the sample size

n/a - this is a pilot study

7b) CONSORT: When applicable, explanation of any interim analyses and stopping guidelines

n/a

8a) CONSORT: Method used to generate the random allocation sequence

"Participants were then randomized using a computer-generated algorithm to the intervention arm or the education control arm. "

8b) CONSORT: Type of randomisation; details of any restriction (such as blocking and block size)

n/a

9) CONSORT: Mechanism used to implement the random allocation sequence (such as sequentially numbered containers), describing any steps taken to conceal the sequence until interventions were assigned

"Participants were then randomized using a computer-generated algorithm to the intervention arm or the education control arm. "

10) CONSORT: Who generated the random allocation sequence, who enrolled participants, and who assigned participants to interventions

"Participants were then randomized using a computer-generated algorithm to the intervention arm or the education control arm. "

11a) CONSORT: Blinding - If done, who was blinded after assignment to interventions (for example, participants, care providers, those assessing outcomes) and how

11a-i) Specify who was blinded, and who wasn't

n/a - given this was a pilot study, study staff were not blinded.

11a-ii) Discuss e.g., whether participants knew which intervention was the "intervention of interest" and which one was the "comparator"

informed consent was given with an explanation of the groups

"At the enrollment events, study staff obtained informed consent, and collected baseline anthropometric and survey measures."

11b) CONSORT: If relevant, description of the similarity of interventions

n/a

12a) CONSORT: Statistical methods used to compare groups for primary and secondary outcomes

"We used chi-square tests and t-tests to examine differences in baseline characteristics between study arms. Similar tests were utilized to describe the average rate of text messaging adherence and the proportion of participants who achieved various thresholds of self-monitoring adherence. We used intent-to-treat analyses, with baseline weight carried forward for missing data. T-tests were conducted to examine absolute weight change, percent weight loss, and BMI change between study arms, and ANOVA was conducted to example weight change across tertiles of intervention adherence. Pearson correlation tests were conducted to examine the overall relationship between text messaging adherence and weight loss. Analyses were conducted using SPSS for Mac (Version 19, Chicago, IL). All tests were two-tailed and an alpha level <0.05 was used to assess statistical significance."

12a-i) Imputation techniques to deal with attrition / missing values

"We used intent-to-treat analyses, with baseline weight carried forward for missing data."

12b) CONSORT: Methods for additional analyses, such as subgroup analyses and adjusted analyses

"We used chi-square tests and t-tests to examine differences in baseline characteristics between study arms. Similar tests were utilized to describe the average rate of text messaging adherence and the proportion of participants who achieved various thresholds of self-monitoring adherence. "

"Pearson correlation tests were conducted to examine the overall relationship between text messaging adherence and weight loss"

RESULTS

13a) CONSORT: For each group, the numbers of participants who were randomly assigned, received intended treatment, and were analysed for the primary outcome

"Figure 2 outlines the study enrollment and retention flow. A total of 149 individuals were deemed eligible via the online screener. Of those, two were ineligible due to BMI and 97 did not attend the enrollment event. Fifty participants were then randomized to either the intervention (n=26) or control (n=24) arm. At 6 months, 90% of participants attended the 6-month follow-up assessment visit. "

Figure 2

13b) CONSORT: For each group, losses and exclusions after randomisation, together with reasons

"At 6 months, 90% of participants attended the 6-month follow-up assessment visit. "

Figure 2

13b-i) Attrition diagram

"Figure 2 outlines the study enrollment and retention flow"

14a) CONSORT: Dates defining the periods of recruitment and follow-up

"Recruitment took place between June and September 2010."

14a-i) Indicate if critical "secular events" fell into the study period

n/a

14b) CONSORT: Why the trial ended or was stopped (early)

n/a

15) CONSORT: A table showing baseline demographic and clinical characteristics for each group

"Participants were on average aged 38.3 ± 8.2 years, obese (BMI 35.8 ± 6.1 kg/m2), and had an average weight of 99.1kg ± 20.0kg (Table 1). Participants were predominantly Black (82%), employed (82%), and college-educated (64%). Approximately one-third of the sample (32%) had an annual income less than $40,000 and 50% were married or living with a partner. There were no significant differences between arms with regard to baseline sociodemographic characteristics (Table 2). "

15-i) Report demographics associated with digital divide issues

"Participants were on average aged 38.3 ± 8.2 years, obese (BMI 35.8 ± 6.1 kg/m2), and had an average weight of 99.1kg ± 20.0kg (Table 1). Participants were predominantly Black (82%), employed (82%), and college-educated (64%). Approximately one-third of the sample (32%) had an annual income less than $40,000 and 50% were married or living with a partner. There were no significant differences between arms with regard to baseline sociodemographic characteristics (Table 2). "

16a) CONSORT: For each group, number of participants (denominator) included in each analysis and whether the analysis was by original assigned groups

16-i) Report multiple "denominators" and provide definitions

"A study database collected and stored text messaging self-monitoring data. Adherence was defined as the proportion of self-monitoring texts received of the number expected (n=167). "

16-ii) Primary analysis should be intent-to-treat

"We used chi-square tests and t-tests to examine differences in baseline characteristics between study arms. Similar tests were utilized to describe the average rate of text messaging adherence and the proportion of participants who achieved various thresholds of self-monitoring adherence. We used intent-to-treat analyses, with baseline weight carried forward for missing data. "

17a) CONSORT: For each primary and secondary outcome, results for each group, and the estimated effect size and its precision (such as 95% confidence interval)

"Among all randomized intervention participants, the daily text messaging self-monitoring adherence rate was 49.0% ± 27.9% (IQR: 27.2% - 78.3%). A majority (57.7%) of participants texted on average at least 3 days each week and 84.6% texted on average at least 2 days per week. "

"At 6 months, using intent-to-treat analyses (n=50), participants randomized to the control arm gained on average 1.14±2.53 kg, while intervention participants lost on average 1.27 ± 6.51 kg [mean difference, (95% CI): -2.41kg (-5.22, 0.39); p=0.09]. This equates to a percent weight loss of 0.97% ±5.35% in the intervention arm, which was marginally higher than a gain of 1.32%±2.77% in the control arm [mean difference, (95% CI): -2.29 (-4.74, 0.16); p=.06]."

17a-i) Presentation of process outcomes such as metrics of use and intensity of use

"Among all randomized intervention participants, the daily text messaging self-monitoring adherence rate was 49.0% ± 27.9% (IQR: 27.2% - 78.3%). A majority (57.7%) of participants texted on average at least 3 days each week and 84.6% texted on average at least 2 days per week. "n/a

17b) CONSORT: For binary outcomes, presentation of both absolute and relative effect sizes is recommended

n/a

18) CONSORT: Results of any other analyses performed, including subgroup analyses and adjusted analyses, distinguishing pre-specified from exploratory

"Similar results were found when we restricted the sample to study completers only (n=45). There were no significant correlations between adherence to text messaging and change in weight or BMI. However, there was a trend towards greater text messaging adherence being associated with greater percent weight loss (r = -.363; p=.08). Similarly, there were no differences in weight outcomes by tertiles of text messaging adherence (data not shown)."

18-i) Subgroup analysis of comparing only users

"Eight participants demonstrated non-usage attrition,[24] requesting to cease intervention participation prior to the end of the trial for a host of reasons (e.g., cost of text messaging, change in phone service, not interested in continued participation). Excluding these eight participants, the adherence rate was 54.2% ±24.7%, and we similarly found no significant predictors of adherence. "

"Similar results were found when we restricted the sample to study completers only (n=45). "

19) CONSORT: All important harms or unintended effects in each group

n/a

19-i) Include privacy breaches, technical problems

n/a - no technical issues

19-ii) Include qualitative feedback from participants or observations from staff/researchers

n/a - no qualitative data collected

DISCUSSION

20) CONSORT: Trial limitations, addressing sources of potential bias, imprecision, multiplicity of analyses

20-i) Typical limitations in ehealth trials

"This study also has some limitations worth mentioning. A few participants (n=8) experienced barriers to participation such as cost of text messaging and disconnected cell phone service, or were no longer interested in participating. Although using personal cell phones enhances the generalizability of the intervention, future studies may consider providing phones and/or text messaging plans.
Although comparable to other eHealth weight control interventions, higher adherence rates are needed in order to produce greater weight losses. This pilot study was low intensity and did not include any contacts with study staff outside the assessment visits. Our main goal was to assess the feasibility of using text messaging for self-monitoring behavioral goals among a predominantly racial/ethnic minority population. To enhance adherence, future studies might benefit from additional intervention components such as coaching or booster DVDs throughout the study period. Similarly, given that this was a pilot study, the small sample size limited our power to assess whether this intervention led to significantly greater weight loss as compared to an education control arm. Future studies should examine the efficacy of this approach with a larger sample size, longer duration, and multiple measures throughout the study period. "

21) CONSORT: Generalisability (external validity, applicability) of the trial findings

21-i) Generalizability to other populations

"Eighty-two percent of our sample was Black women, which is a group typically underrepresented in weight control research; although a strength of our study, the findings may not generalize to other populations and settings. "

21-ii) Discuss if there were elements in the RCT that would be different in a routine application setting

"To enhance adherence, future studies might benefit from additional intervention components such as coaching or booster DVDs throughout the study period."

"Our pilot study used text messaging to collect self-monitoring data on diet and physical activity behaviors, but we did not gather data on body weight or include any type of coaching support. This may have affected the weight losses achieved, as body weight self-monitoring has been shown to be effective in the absence of self-monitoring of other behaviors."

22) CONSORT: Interpretation consistent with results, balancing benefits and harms, and considering other relevant evidence

22-i) Restate study questions and summarize the answers suggested by the data, starting with primary outcomes and process outcomes (use)

"We found within this pilot study that daily text messaging for behavioral self-monitoring is both feasible and positively perceived. About half of participants were fully adherent to daily self-monitoring via text message during the 6-month study and 84% stayed active in the intervention throughout the study period. In contrast to previous studies that included paper-based self-monitoring modes,[5] a majority of participants felt that text messaging was helpful, easy, and important for achieving their behavior change goals. Text messaging has major advantages relative to other approaches. It has high familiarity and a greater potential for broad reach, particularly among racial/ethnic minority populations. Despite the intervention having only a marginal effect on weight change, these results indicate that text messaging may be a viable way to collect self-monitoring data and deliver intervention feedback and skills training. "

22-ii) Highlight unanswered new questions, suggest future research

"This study has several strengths. Eighty-two percent of our sample was Black women, which is a group typically underrepresented in weight control research. The goal of this study was to test the feasibility of text messaging for self-monitoring via a low-intensity weight loss intervention among an understudied population. We more closely isolated the impact of text messaging self-monitoring along with feedback with a control arm that received comparable group information sessions and the use of a pedometer."

"To enhance adherence, future studies might benefit from additional intervention components such as coaching or booster DVDs throughout the study period. Similarly, given that this was a pilot study, the small sample size limited our power to assess whether this intervention led to significantly greater weight loss as compared to an education control arm. Future studies should examine the efficacy of this approach with a larger sample size, longer duration, and multiple measures throughout the study period. "

Other information

23) CONSORT: Registration number and name of trial registry

"Clinical trials.gov information: NCT00939081"

24) CONSORT: Where the full trial protocol can be accessed, if available
n/a - we don't have the full protocol published elsewhere
25) CONSORT: Sources of funding and other support (such as supply of drugs), role of funders
"Funding/Support: This trial is funded by grant K22CA126992 awarded to Dr. Gary Bennett"
X26-i) Comment on ethics committee approval
"The Duke University Institutional Review Board approved this study. "
x26-ii) Outline informed consent procedures
not online - it was conducted in-person

"At the enrollment events, study staff obtained informed consent, and collected baseline anthropometric and survey measures. "
X26-iii) Safety and security procedures
n/a - pilot study with limited potential for harms
X27-i) State the relation of the study team towards the system being evaluated
"Conflicts of Interest: None declared"
